# Dietary Supplementation of EGF Ameliorates the Negatively Effects of LPS on Early-Weaning Piglets: From Views of Growth Performance, Nutrient Digestibility, Microelement Absorption and Possible Mechanisms

**DOI:** 10.3390/ani11061598

**Published:** 2021-05-28

**Authors:** Junjing Xue, Liang Xie, Bo Liu, Liyuan Zhou, Yajun Hu, Kolapo Matthew Ajuwon, Rejun Fang

**Affiliations:** 1 College of Animal Science and Technology, Hunan Agricultural University, No. 1 Nongda Road, Furong District, Changsha 410128, China; Xuejunjing@stu.hunau.edu.cn (J.X.); xl@stu.hunau.edu.cn (L.X.); xb19940608@163.com (B.L.); zly0430@stu.hunau.edu.cn (L.Z.); Huyajun@stu.hunau.edu.cn (Y.H.); 2 Hunan Co-Innovation Center of Animal Production Safety, Changsha 410128, China; 3 Department of Animal Sciences, Purdue University, West Lafayette, IN 47907-2054, USA; kajuwon@purdue.edu

**Keywords:** early weaning piglets, different levels of LPS and EGF, growth performance, nutrition digestibility, microelement absorption, microelement transport-relative gene

## Abstract

**Simple Summary:**

This study aims to investigate how epidermal growth factor (EGF) attenuates the effect of lipopolysaccharide (LPS) on the growth performance, nutrient digestibility, microelement absorption of early-weaned pigs. A total of 48 early weaned piglets were randomly distributed to four groups consisting of a 2 × 2 factorial design. The main factors were the level of LPS (H_LPS_ = high LPS: 100 μg/kg body weight; Z_LPS_ = low LPS: 0 μg/kg body weight) and EGF (H_EGF_ = high EGF: 2 mg/kg diet; Z_EGF_ = low EGF: 0 mg/kg diet). Each group had four replicates and each replicate consisted of three piglets. The results showed that H_LPS_ level decreased the growth performance and the apparent digestibility of crude fat, while H_EGF_ level increased the average daily feed intake. The concentration of most microelements in the gastrointestinal tract chyme and feces were increased by H_LPS_ level and decreased by H_EGF_ level. The expression levels of most microelement transport-relative genes in the mucosa of gastrointestinal tissues were decreased by H_LPS_ level and increased by H_EGF_ level. In conclusion, dietary EGF could attenuate the negative effect of LPS exposure on the apparent digestibility of crude fat and microelement absorption through changing the expression levels of microelement transport-relative genes. EGF can be used as an additive to increase the essential trace elements absorption in the early weaning piglets.

**Abstract:**

Epidermal growth factor (EGF) plays an important role in nutrients absorption. However, whether it can be an effective additive to improve the growth performance and nutrients absorption in lipopolysaccharide (LPS) challenged early weaning piglets is still unknown. A 14-days trial was conducted to investigate how EGF attenuates the effect of LPS on the growth performance, nutrient digestibility, microelement absorption of early-weaned pigs, and study the underlying mechanism. A total of 48 early weaned piglets, aged 25 days, were randomly distributed to four groups (control, EGF, LPS and EGF + LPS groups) consisting of a 2 × 2 factorial design. The main factors were the level of LPS (H_LPS_ = high LPS: 100 μg/kg body weight; Z_LPS_ = low LPS: 0 μg/kg body weight) and EGF (H_EGF_ = high EGF: 2 mg/kg diet; Z_EGF_ = low EGF: 0 mg/kg diet). Each group had four replicates and each replicate consisted of three piglets. The results showed that piglets injected with H_LPS_ level significantly decreased the average daily gain (ADG), and significantly increased the feed conversion ratio (FCR) compared with the piglets injected with Z_LPS_ level, while piglets fed H_EGF_ level significantly increased the average daily feed intake (ADFI) compared with the piglets fed Z_EGF_ level (*p* < 0.05). Piglets injected with H_LPS_ level significantly decreased the apparent digestibility of crude fat compared with the piglets injected with Z_LPS_ level (*p* < 0.05). Piglets injected with H_LPS_ level significantly increased the concentration of most microelements in the gastrointestinal tract chyme and feces, and significantly decreased the expression levels of most microelement transport-relative genes in the mucosa of gastrointestinal tissues compared with the piglets injected with Z_LPS_ level (*p* < 0.05). Piglets fed H_EGF_ level significantly decreased the concentration of microelement in the gastrointestinal tract chyme and feces, and significantly increased the expression levels of the microelement transport-relative genes in the mucosa of gastrointestinal tissues compared with the piglets fed Z_EGF_ level (*p* < 0.05). In conclusion, dietary EGF could attenuate the negative effect of LPS exposure on the apparent digestibility of crude fat and microelement absorption of early-weaning piglets. EGF and LPS influenced the absorption of essential trace element through changing the expression levels of microelement transport-relative genes in the mucosa of gastrointestinal tissues. In the early weaning piglets, EGF can be used as an additive to increase the essential trace elements absorption.

## 1. Introduction

Essential trace elements are the indispensable nutrients for animals, and especially Cu, Fe, Zn, and Mn are required for the normal growth, development, and many physiological functions in animals [1,2,3,4]. Cu is a part of Cu-transporting P-type ATPase and Cu/Zn superoxide dismutase [5]. Fe as the part of hemoglobin and myoglobin plays an important role in delivering the oxygen, and it also plays a vital role in the host immunity [6]. Zn takes part in the growth, oxidation resistance and immunity [7]. Mn as the part of phosphoenolpyruvate carboxykinase takes part in the gluconeogenesis, and it is related to the neuronal health [8].

Pig (*Sus scrofa*) is one of the most raised animals in the world. Piglets are weaned early to increase the reproductive performance of the sow and to reduce pathogen transmission [9]. However, as the digestive system of piglets is immature, early weaning will lead to maldigestion [10]. Meanwhile, because of rapid and dramatic change of the living environment and exposure to the bacteria [11], early weaning piglets easily suffer from stress, which reduces the growth performance and feed intake [12] and decreases the nutrient digestibility through digestive disorders [13]. It leads to the resources waste and environment pollution and limits the sustainable development of animal husbandry. Thus, it is urgent to look for an effective additive to relieve early weaning stress and improve the absorption of nutrition. The absorption of nutrition is closely related to the intestinal health, however, early weaning stress increases the intestinal permeability of piglets which has a negative effect on the absorption of nutrients [14]. Lipopolysaccharide (LPS) is the primary component of Gram-negative bacteria outer cell walls [15] and it can induce severe bacterial diarrhea, apoptosis [16], inflammatory responses [17], intestinal barrier damage [18], and then inhibits the growth performance and decreases the nutrients absorption of the animal [19]. Due to its good repeatability, the LPS stress mode is widely used in research.

Many of growth factors exist in milk, such as insulin, nerve growth factor (NGF), and epidermal growth factor (EGF) which can improve the intestinal development of piglets and thus improve their growth performance [20]. Early weaning prevents the supply of those growth factors from milk to piglets. Interestingly, EGF is one of the most abundant growth factors in milk [21,22], which indicates its important role for young mammals. EGF was first isolated by Dr. Cohen from the mouse (*Mus musculus*) submaxillary gland in 1962 [23]. It is a polypeptide comprising 53 amino acids [24]. It is found in many body fluids such as the milk, blood, saliva, and intestinal fluid [25], and it plays important roles in the regulation of cell growth, proliferation, apoptosis and tumorigenesis [26,27,28]. Previous studies showed that EGF could improve the growth performance of broiler chicks (*Gallus gallus*) [27] and rats (*Rattus norvegicus*) [29]. Dietary EGF can augment the intestinal length and villus height by activating the phosphatidylinositol-3-kinases/protein-serine-threonine kinase (PI3K/AKT) and RAS/mitogen-activated protein kinase (RAS/MAPK) signaling pathways [30,31]. Meanwhile, EGF can also promote the proliferation of goblet cells [10] and increase the activity of digestive enzymes in the intestine [32]. However, the effect of EGF on growth performance and nutrients absorption in LPS challenged early-weaning pigs is unclear. Whether it can be added as an effective additive in the feed of early weaning piglets is still unknown. In this experiment, a model of LPS stress was established to examine how EGF attenuates the effect of LPS on the growth performance, nutrient digestibility, microelement absorption of early-weaned pigs, and study the underlying mechanism.

## 2. Materials and Methods

### 2.1. Experimental Design

A total of 48 Duroc × Landrace × Large White early weaned piglets (castrated male pigs, average initial weight was 7.84 ± 0.30 kg), aged 25 days, were randomly distributed among four groups (control, EGF, LPS and EGF + LPS groups) which consisted of a 2 × 2 factorial design. Each group had four replicates and each replicate consisted of three piglets. The main factors were the level of LPS (H_LPS_ = high LPS: 100 μg/kg body weight; Z_LPS_ = low LPS: 0 μg/kg body weight) and EGF (H_EGF_ = high EGF: 2 mg/kg diet; Z_EGF_ = low EGF: 0 mg/kg diet). Piglets in the LPS and EGF + LPS groups were intraperitoneally injected with the 100 μg/kg body weight LPS (Sigma-Aldrich, Saint Louis, MO, USA) at 7 and 15 days during the experiment [33]. Meanwhile, the control and EGF groups were injected with the corresponding volume physiological saline (Nanjing Jiancheng Biotechnical Institute, Nanjing, China). The control and LPS groups were fed the basal diet (diet 1) which met the nutrient requirements of pigs according to NRC 2012 (Table 1). The piglets in the EGF and EGF + LPS groups were fed the basal diet supplemented with 2 mg/kg EGF (diet 2, Peprotech, Rocky Hill, CT, USA). The experiment lasted for 14 days and the pigs had ad libitum access to feed and water during this period. The humidity ranged from 50% to 70%, and the temperature ranged from 18 to 22 °C. The pigs were fasted for 24 h and were weighed in the morning at 1 day and 15 days during the experiment, and feed intake was recorded every day. At the end of the trial, initial body weight (IBW), final body weight (FBW), average daily feed intake (ADFI), average daily gain (ADG), and feed conversion ratio (FCR, feed/gain) were calculated.

### 2.2. Sample Collection

Feces were collected from days 11 to 14 during the trial and were stored at −20 °C. At the end of the experiment, all pigs were slaughtered 4 h after the final injection of LPS. Before slaughter, all piglets were euthanized with Zoletil (active compound: tiletamine and zolazepam, Virbac, Beijing, China) at 15 mg/kg body weight. The chyme samples from the stomach, jejunum and ileum were collected and immediately frozen at −20 °C. The stomach, duodenum, jejunum and ileum samples were washed with saline solution, and then the mucosa of these samples was collected by glass slide and immediately frozen at −80 °C for Q-RT-PCR analysis.

### 2.3. Nutrient Digestibility and Essential Microelements Concentration

The diet, feces and chyme samples were dried at 105 °C. Then, they were ground into a fine powder and passed through a 40 μm mesh. Gross energy, crude protein, crude fat, crude fiber, and P were tested according to the methods of the Association of Official Analytical Chemists International, 2007. The digestibility of nutrients was calculated as follows:

Digestibility (%) = 100 − (I_d_ ÷ I_s_) × (N_s_ ÷ N_d_) × 100%, (1)

where the I_d_ and I_s_ are the concentration of the acid-insoluble ash in the diet and the feces, respectively, and N_s_ and N_d_ are the concentration of the nutrient in the feces and the diet, respectively.

Samples of diet, feces and chyme were digested in the concentrated nitric acid and perchloric acid mixture solution (the addition ratio of concentrated nitric acid and perchloric acid was 4:1) to dissolve the Cu, Fe, Zn and Mn (GB/T 23942-2009), and the concentration was analyzed by electron coupled plasma atomic emission spectrum (Ke Jie Instrument Limited Company, Nanjing, China).

### 2.4. Quantitative Real-Time PCR (Q-RT-PCR) Analysis

The relative expression levels of zrt-irt-like protein 4 (Zip4), zrt-irt-like protein 7 (Zip7), zinc transporter 1 (ZnT1), zinc transporter 4 (ZnT4), copper transport protein 1 (Ctr1), cytochrome c oxidase copper chaperone 17 (Cox17), antioxidant 1 (Atox1), copper-transporting P-type 7A (ATP7A), copper-transporting P-type 7B (ATP7B), copper chaperone for superoxide dismutase (CCS), divalent metal transporter 1 (DMT1), cytochrome b (CYTB), hephaestin (Hp), and transferrin (Tf) in the mucosa of the stomach, duodenum, jejunum and ileum were detected by Q-RT-PCR. The primers (Sangon Biotech, Shanghai, China) used are listed in Supplementary Table S1. The glyceraldehyde-3-phosphate dehydrogenase (GAPDH) gene was chosen as the reference gene for sample normalization. Total RNA from the intestinal tissue was extracted using the TRIzol reagent (Invitrogen, Carlsbad, CA, USA). The integrity of each RNA sample was estimated by 1% agarose gel electrophoresis (Sangon Biotech, Shanghai, China). The cDNA was synthesized using a SMART cDNA Synthesis Kit (Clontech Laboratories, Palo Alto, CA, USA) by following the manufacturer’s protocol. Q-RT-PCR reactions were carried out in a BIO-RAD CFX96 touch Q-PCR system (Applied Biosystems, Foster City, CA, USA) in 20 μL volumes that contained the following components: 10 μL of SYBR Green Mix (Takara, Changsha, China), 2 μL cDNA (1000 ng·μL^−1^), 0.4 μL of each primer (10 mM) and 7.2 μL dH_2_O, followed by 40 cycles of 95 °C for 30 s, 55 °C or 58 °C for 30 s, and 72 °C for 30 s. Finally, a melt curve analysis was used to detect the single product (temperature from 65 to 95 °C). All samples were tested in triplicate. The 2^−ΔΔCT^ method was used to analyze the relative expression level. The standard curve was obtained by using 5-fold serial dilutions of cDNA (in triplicate), and the amplification efficiencies of all primes ranged from 0.90 to 1.00.

### 2.5. Statistical Analysis

The experimental design was a 2 × 2 factorial design while the main factors were the level of LPS and EGF. Data were analyzed by 2-way ANOVA using SPSS 23.0 (SPSS. Inc., Chicago, IL, USA), which included the main effects of LPS level, EGF level and their interaction (LPS level × EGF level). Tukey’s multiple range test was used to analyze the differences. All data were further subjected to one-way ANOVA. When overall differences were significant, the differences were tested by Duncan’s multiple-range test (SPSS 22.0). The data about the concentration of essential microelements in the diets were subjected to independent-samples T test (SPSS 22.0). The level of significance was set at *p* < 0.05. The results are presented as the mean values and standard error of mean (SEM).

## 3. Results

### 3.1. Cu, Fe, Zn, and Mn Concentration in Diets

The concentration of essential microelements in diets 1 and 2 are shown in Table 2. There were no significant differences of Cu, Fe, Zn, and Mn concentration between diet 1 and diet 2 (*p* > 0.05).

### 3.2. Growth Performance

The effect of LPS and EGF levels on growth performance are shown in Table 3. The LPS level affected ADG and FCR of piglets, and EGF level affected ADFI (*p* < 0.05). The LPS and EGF levels displayed a significant interaction effect on ADFI (*p* < 0.05). Piglets injected with H_LPS_ level significantly decreased the ADG and significantly increased the FCR compared with the piglets injected with Z_LPS_ level (*p* < 0.05). The ADFI of piglets fed H_EGF_ level was 12% higher than the piglets fed Z_EGF_ level (*p* < 0.05). The lowest ADFI was observed in the control group, which significantly differed from the other three groups (*p* < 0.05)

### 3.3. Nutrient Apparent Digestibility

Except for the apparent digestibility of crude fat, no differences were observed in the nutrient apparent digestibility, and there were no interactions between LPS and EGF levels regarding the apparent digestibility (*p* > 0.05, Table 4). Compared with Z_LPS_ level, H_LPS_ level significantly decreased the apparent digestibility of crude fat (*p* < 0.05). The lowest apparent digestibility of crude fat was observed in the LPS group, which was significantly differed from the other groups (*p* < 0.05), and there was no significant difference between the EGF + LPS and control groups (*p* > 0.05).

### 3.4. Concentration of Cu, Fe, Zn, Mn in the Gastrointestinal Chyme and Feces

The concentration of Cu, Fe, Zn, Mn in the gastrointestinal chyme and feces are shown in Table 5. The present study revealed significantly interactions between the EGF and LPS levels regarding the Cu concentration in the stomach, jejunum and ileum chyme (*p* < 0.05), and there was no interaction in the feces (*p* > 0.05). Piglets injected with H_LPS_ level decreased the Cu concentration in the ileum chyme and increased the Cu concentration in the jejunum chyme compared the piglets injected with Z_LPS_ level (*p* < 0.05). The Cu concentration in the jejunum, ileum chyme and feces of piglets fed H_EGF_ level were 18%, 55%, and 28% lower than those of piglets fed Z_EGF_ level, respectively (*p* < 0.05). The LPS group had a significantly greater Cu concentration in the jejunum chyme compared with the other groups (*p* < 0.05), while there was no significant difference between the EGF + LPS and control groups (*p* > 0.05). The control and LPS groups had significantly greater Cu concentration in the ileum chyme compared with the EGF and EGF + LPS groups (*p* < 0.05).

The LPS and EGF levels displayed a significant interaction effect on the Fe concentration in the feces (*p* < 0.05). Piglets injected with H_LPS_ level decreased the Fe concentration in the ileum chyme and increased the Fe concentration in the jejunum chyme and feces compared with the piglets injected with Z_LPS_ level (*p* < 0.05). The Fe concentration in the ileum chyme and feces of piglets fed H_EGF_ level were 35%, and 31% lower than those of piglets fed Z_EGF_ level, respectively (*p* < 0.05). In the feces, the LPS group had a significantly greater Fe concentration compared with the other groups (*p* < 0.05), and the EGF + LPS group had a significantly lower Fe concentration compared with the control group (*p* < 0.05).

The LPS and EGF levels displayed significant interactions on the Zn concentration in the stomach and ileum chyme, and feces (*p* < 0.05). The Zn concentration in the jejunum, ileum chyme and feces of piglets injected with H_LPS_ level were 12%, 53%, and 19% higher than those of piglets fed Z_LPS_ level, respectively (*p* < 0.05). The Zn concentration in the jejunum, ileum chyme and feces of piglets fed H_EGF_ level were 12%, 68%, and 28% lower than those of piglets fed Z_EGF_ level, respectively (*p* < 0.05). In the ileum chyme, a significantly greater Zn concentration was observed in the LPS group, which significantly differed from the other groups (*p* < 0.05), and the EGF + LPS group had a significantly lower Zn concentration compared with the control group (*p* < 0.05). In the feces, the LPS group had a significantly greater Zn concentration compared with the other groups (*p* < 0.05), while there was no significant difference between the EGF + LPS and control groups (*p* > 0.05).

Piglets injected with H_LPS_ level significantly increased the Mn concentration in the stomach chyme and feces compared with the piglets injected with Z_LPS_ level (*p* < 0.05). The Mn concentration in the jejunum, ileum chyme and feces of piglets fed H_EGF_ level were 29%, 15%, and 27% lower than those of piglets fed Z_EGF_ level, respectively (*p* < 0.05). In the stomach chyme, the LPS and EGF + LPS groups had significantly greater Mn concentration compared with the control and EGF groups (*p* < 0.05). In the feces, the LPS group had a significantly greater Mn concentration compared with the other groups (*p* < 0.05), and the EGF + LPS group had a significantly lower Mn concentration compared with the control group (*p* < 0.05).

### 3.5. Expression of Cu Transport-Relative Genes in the Mucosa of the Gastrointestinal Tissues

As shown in Table 6, the expression levels of the Cu transport-related genes in the mucosa from the gastrointestinal tissues were affected by LPS and EGF levels (*p* < 0.05). In the stomach, the LPS and EGF levels displayed significant interaction effects on the expression levels of Cox17, Atox1, ATP7A, and ATP7B (*p* < 0.05). Piglets injected with H_LPS_ level significantly decreased the expression levels of Atox1 and ATP7B compared with the piglets injected with Z_LPS_ level (*p* < 0.05), and piglets supplied with H_EGF_ level significantly increased the expression levels of Ctr1, Cox17, Atox1, ATP7A, and ATP7B compared with the piglets supplied with Z_EGF_ level (*p* < 0.05). The LPS and EGF + LPS groups had a significantly lower expression level of Atox1 compared with the control and EGF groups (*p* < 0.05), whereas there was no significant difference between the EGF + LPS and LPS groups (*p* > 0.05). The LPS group had a significantly lowest expression level of ATP7A compared with the other groups (*p* < 0.05), and EGF + LPS group had a significantly higher expression level compared with the LPS and control groups (*p* < 0.05).

In the duodenum, there were significant interactions between LPS and EGF levels in the expression levels of Cox17, Atox1, and ATP7B (*p* < 0.05). Compared with the piglets injected with Z_LPS_ level, piglets injected with H_LPS_ level significantly decreased the expression levels of Cox17, ATP7A, ATP7B, and CCS (*p* < 0.05). Piglets supplied with H_EGF_ level significantly increased the expression levels of Cox17, ATP7A, and ATP7B compared with the piglets supplied with Z_EGF_ level (*p* < 0.05). The LPS group had the significantly lowest expression level of Cox17 compared with the other groups (*p* < 0.05), whereas there was no significant difference between the EGF + LPS and control groups (*p* > 0.05). The LPS group had a significantly lower expression level of ATP7A compared with the control and EGF groups (*p* < 0.05), while there was no significant difference between the EGF + LPS and control groups, or between the EGF + LPS and LPS groups (*p* > 0.05).

In the jejunum, the LPS and EGF levels displayed a significant interaction effect on the expression level of CCS (*p* < 0.05). The H_LPS_ level significantly decreased the expression levels of Ctr1, Atox1, and CCS compared with the Z_LPS_ level, and the H_EGF_ level significantly increased the expression levels of Ctr1, Atox1, ATP7A, ATP7B, and CCS compared with the Z_EGF_ level (*p* < 0.05). The LPS group had the significantly lowest expression levels of Ctrl and Atox1 compared with the other groups (*p* < 0.05), while there was no significant difference between the EGF + LPS and control groups (*p* > 0.05).

In the ileum, there was a significant interaction between LPS and EGF levels in the expression level of Atox1 (*p* < 0.05). Piglets injected with H_LPS_ level significantly decreased the expression levels of Cox17, ATP7B, and CCS compared with the piglets injected with Z_LPS_ level (*p* < 0.05), and piglets supplied with H_EGF_ level significantly increased the expression level of Atox1 compared with the piglets supplied with Z_EGF_ level (*p* < 0.05). The LPS and EGF + LPS groups had significantly lower expression levels of Cox17, ATP7B, and CCS compared with the control and EGF groups (*p* < 0.05), whereas there was no significant difference between the EGF + LPS and LPS groups (*p* > 0.05). The LPS group had the significantly lowest expression level of Atox1 compared with the other groups (*p* < 0.05), and the EGF + LPS group had a significantly greater expression level compared with the control group (*p* < 0.05).

### 3.6. Expression of Fe Transport-Relative Genes and DMT1 Gene in the Mucosa of the Gastrointestinal Tissues

The expression levels of Fe transport-related genes and DMT1 gene in the mucosa of the gastrointestinal tissues are shown in Table 7. In the stomach, the LPS and EGF levels displayed a significant interaction effect on the expression level of CYTB (*p* < 0.05). Piglets injected with H_LPS_ level significantly decreased the expression level of Tf compared with the piglets injected with Z_LPS_ level (*p* < 0.05), and piglets supplied with H_EGF_ level significantly increased the expression levels of CYTB, Hp, Tf, and DMT1 compared with the piglets supplied with Z_EGF_ level (*p* < 0.05).

In the duodenum, there were significant interactions between LPS and EGF levels in the expression levels of CYTB and DMT1 (*p* < 0.05). Compared with the piglets injected with Z_LPS_ level, piglets injected with H_LPS_ level significantly decreased the expression levels of Tf and DMT1 (*p* < 0.05). Supply with H_EGF_ level significantly increased the expression levels of CYTB and DMT1 compared with the piglets supplied with Z_EGF_ level (*p* < 0.05). The LPS and EGF + LPS groups had significantly lower expression levels of Tf and DMT1 compared with the control and EGF groups (*p* < 0.05), whereas there was no significant difference between the EGF + LPS and LPS groups (*p* > 0.05).

In the jejunum, the H_LPS_ level significantly decreased the expression levels of CYTB, Tf, and DMT1 compared with the Z_LPS_ level, and the H_EGF_ level significantly increased the expression levels of Hp and DMT1 compared with the Z_EGF_ level (*p* < 0.05). The LPS and EGF + LPS groups had significantly lower expression level of CYTB compared with the control and EGF groups (*p* < 0.05), whereas there was no significant difference between the EGF + LPS and LPS groups (*p* > 0.05). The LPS group had a significantly lower expression level of DMT1 compared with the control and EGF groups (*p* < 0.05), while there was no significant difference between the EGF + LPS and control groups, or between the EGF + LPS and LPS groups (*p* > 0.05).

In the ileum, there were significant interactions between LPS and EGF levels in the expression levels of Hp and DMT1 (*p* < 0.05). Piglets injected with H_LPS_ level significantly decreased the expression level of DMT1 compared with the piglets injected with Z_LPS_ level (*p* < 0.05), and piglets supplied with H_EGF_ level significantly increased the expression levels of CYTB and Hp compared with the piglets supplied with Z_EGF_ level (*p* < 0.05). The LPS group had the significantly lowest expression level of Hp compared with the other groups (*p* < 0.05), and the EGF + LPS group had a significantly greater expression level compared with the control group (*p* < 0.05). The LPS group had a significantly lower expression level of DMT1 compared with the other groups (*p* < 0.05), but there was no significant difference between the EGF + LPS and control groups (*p* > 0.05). 

### 3.7. Expression of Zn Transport-Relative Genes in the Mucosa of the Gastrointestinal Tissues

The expression levels of Zn transport-related genes in the mucosa from the gastrointestinal tissues are shown in Table 8. The present study revealed significant interactions between EGF and LPS levels regarding the expression level of Zip4 in the stomach, and the expression levels of Zip7 in the stomach and ileum (*p* < 0.05). Injected H_LPS_ level significantly decreased the expression levels of Zip4 and Zip7 in the stomach and ileum compared with the Z_LPS_ level (*p* < 0.05) and supplied H_EGF_ level significantly increased the expression level of Zip4 in the stomach, jejunum and ileum compared with the Z_EGF_ level. The LPS group had a significantly lower expression level of Zip4 in the ileum compared with the other groups (*p* < 0.05), whereas there was no significant difference between the EGF + LPS and control groups (*p* > 0.05). The LPS and EGF + LPS groups had significantly lower expression levels of Zip7 in the stomach and ileum compared with the control group (*p* < 0.05), while there was no significant difference between the LPS and EGF + LPS groups (*p* > 0.05). For ZnT1, injected H_LPS_ level significantly increased the expression level of it in the stomach compared with the Z_LPS_ level (*p* < 0.05), and supplied H_EGF_ level significantly increased the expression level of it in the stomach compared with Z_EGF_ level (*p* < 0.05).

## 4. Discussion

Recently, the application of EGF has received increasing amounts of attention due to its positive impacts on animals [26,27,28]. Previous studies had shown that LPS significantly decreased the ADG and ADFI of weaned piglets [34], and EGF could increase body weight gain of broiler chickens and early-weaned mice [27,35], and increase gain/feed of early-weaned pigs [36]. Our results indicated that injected H_LPS_ level significantly decreased the ADG and significantly increased the FCR, and dietary H_EGF_ level significantly increased the ADFI of early-weaned piglets, which were in agreement with the previous studies. Our results also indicated that injected H_LPS_ level significantly decreased the apparent digestibility of crude fat. The changes of growth performance induced by LPS was related to the changes of nutrients absorption. LPS leads to partial loss and sloughing of ileal villi and decreases the intestinal barrier function in mice [37]. LPS also increases the intestinal epithelial cell permeability [38]. LPS reduced the apparent digestibility maybe through reducing the intestinal health. Previous study had showed that dietary EGF had no significant influence on the apparent digestibility of crude protein, gross energy and P [39]. Our results also indicated that dietary H_EGF_ level had no significant influence on the apparent digestibility, which was in agreement with the previous studies.

Indispensable microelements take part in the regulation of the body physiological functions, such as participating in the redox active [1,2,3,4], oxygen transport, DNA biosynthesis [40], cellular signal recognition [41,42], and nutrients metabolism [43,44]. A higher concentration of microelements in the gastrointestinal chyme and feces means a lower absorption level. Cu as a cofactor plays an essential role in redox-active, pigmentation, oxidative phosphorylation and neuropeptide biogenesis [45,46]. Our results showed that injected H_LPS_ level increased the concentration of Cu in the jejunum chyme, and dietary H_EGF_ level decreased the concentration of Cu in the jejunum, ileum chyme, and feces. ATP7A, ATP7B, Cox17, Ctr1, Atox1, and CCS genes are Cu transport-related genes. Ctr1 is a major Cu extracellular uptake protein and involved Cu transport across membranes [47]. Atox1, CCS, and Cox17 are metallochaperones: Cox17 transport Cu to the mitochondria, and CCS transport Cu to combine the SOD in the cytoplasm and mitochondria, and Cox17 transport Cu to combine the ATP7A and ATP7B [48]. ATP7A and ATP7B are P-type Cu-ATPases which transport Cu to the ceruloplasmin and lysyl oxidase and take part in the Cu exportation from the cell [49]. Our results showed that in the mucosa of gastrointestinal tissues, injected H_LPS_ level decreased the expression levels of the ATP7A, ATP7B, Cox17, Ctr1, Atox1, and CCS genes, while dietary H_EGF_ level increased the expression levels of these genes. These results explained how LPS and EGF regulated the absorption of Cu in the gastrointestinal tissues. However, injected H_LPS_ level decreased the concentration of Cu in the ileum, and the underlying reason needs further analysis.

Fe is the transporter of oxygen, and it takes part in the redox reaction, electron transport, cell growth, and energy production [50,51]. Our results showed that injected H_LPS_ level increased the concentration of Fe in the jejunum chyme and feces, while dietary H_EGF_ level decreased the concentration of Fe in the ileum chyme and feces. CYTB, Tf and HP are Fe transport-related genes. CYTB and HP are oxidoreductases: CYTB as ferric reductase changes ferric iron to ferrous iron [52], while Hp is expressed in the enterocyte and oxidizes ferrous iron [50,53]. CYTB co-operate with DMT1 to transfer Fe from the duodenal lumen to the enterocyte, while Hp co-operate with ferroportin to transfer Fe from the basolateral membrane to the systemic circulation [50]. Tf is synthesized almost in the liver [54], and it can regulate iron homeostasis and erythropoiesis [55]. As an important iron carrier in the blood [53], Tf delivers iron to the tissues [54]. Our results showed that in the mucosa of gastrointestinal tissues injected H_LPS_ level decreased the expression levels of the CYTB and Tf genes, and dietary H_EGF_ level increased the expression levels of the CYTB, Tf and HP genes. Consequently, LPS and EGF affected the absorption of Fe in the gastrointestinal tissues by regulating the expression levels of Fe transport-related genes.

Mn as enzymatic cofactors or structural centers takes part in a plethora of biological processes, such as glycosylation, signal transduction, phosphorylation, and hydrolysis [56,57]. It also takes part in the host immune system [58]. Our results showed that injected H_LPS_ level increased the concentration of Mn in the stomach chyme and feces, while dietary H_EGF_ level decreased the concentration of Mn in the jejunum and ileum chyme and feces. DMT1 is a multiple divalent metals transport gene, and it can transfer the Cu, Mn, Zn [59]. Our results showed that in the mucosa of gastrointestinal tissues injected with H_LPS_ level downregulated the expression level of the DMT1 gene, while dietary H_EGF_ level upregulated the expression level of the DMT1 gene. That was the one reason why the LPS and EGF changed the absorption of Cu, Mn, and Zn in the piglets.

Zn is the cofactor for many enzymes and takes part in many biological processes [60], and it plays the important roles in protein synthesis, growth, and immunity [61]. Our results showed that in the jejunum and ileum chyme, and feces, injected H_LPS_ level increased the concentration of Zn, while dietary H_EGF_ level decreased the concentration of Zn. In the body of vertebrates two kinds of zinc transporter family proteins exist, ZIP and ZnT family [62]. Zip4, Zip7 and ZnT1 are the Zn transport-related genes. Zip4, Zip7 belong to the ZIP family and take part in the import of Zn to the cytoplasm. Zip4 is a primary importer for the absorption of Zn in the enterocyte, and it can transfer Zn from intestine lumen to the epithelial cells [63]. Zip7 exists in the membrane of endoplasmic reticulum and Golgi apparatus, and transfers Zn to the cytosol [64]. ZnT1 belongs to the ZnT family, which predominantly localizes in the basolateral membrane [65]. In the intestinal epithelial cells, it takes part in the export of Zn from the cytoplasm to the portal vein [65]. Our results showed that in the mucosa of gastrointestinal tissues, injected H_LPS_ level decreased the expression levels of the Zip4, Zip7 and ZnT1 genes, while dietary H_EGF_ level increased the expression levels of the Zip4 and ZnT1 genes. It is implied that the bioavailability of Zn was affected by LPS and EGF through regulating the expression of Zn transport-related genes.

## 5. Conclusions

In conclusion, the present findings suggested that intraperitoneal injection with H_LPS_ level increased the FCR, and decreased the ADG, apparent digestibility of crude fat, and absorption of Cu Fe, Zn, Mn in the early-weaned pigs. Dietary EGF could reduce the adverse effect of LPS exposure on apparent digestibility of crude fat and microelement absorption of early-weaning piglets. EGF and LPS influenced the absorption of essential trace element through changing the expression levels of Zip4, Zip7, ZnT1, Ctr1, Atox1, CCS, Cox17, ATP7A, ATP7B, DMT1, CYTB, Hp and Tf genes in the mucosa of gastrointestinal tissues. Hence, EGF can be used as an additive to increase the essential trace elements absorption in the early weaning piglets.

## Figures and Tables

**Table 1:** Composition of the basal diet and nutrition level (dry matter).

Ingredient	Content, %	Nutrient Level ^2^	Content
Corn	63.70	Digestible energy, MJ/kg	14.90
Squeezed soybean meal	16.00	Crude protein, %	19.59
Expanded soybean	8.00	Lys, %	1.56
Fish meal	4.50	Met + Cys, %	0.88
Whey powder	2.00	Ca, %	0.86
Glucose	2.00	Available P, %	0.45
Limestone	0.78	Total P, %	0.61
CaHPO4	1.30	Crude fat, %	4.59
Lys	0.35	Crude fiber, %	3.65
Met	0.07		
Thr	0.06		
NaCl	0.24		
Premix ^1^	1.00		
Total	100		

^1^ The premix provided per kilogram of complete feed: vitamin A, 10,000 IU; vitamin D3, 1500 IU; vitamin E, 60 mg; vitamin K3, 3 mg; vitamin B1, 1.8 mg; vitamin B12, 0.024 mg; riboflavin, 6 mg; folic acid, 0.3 mg; biotin, 4.5 mg; nicotinic acid, 24 mg; D-pantothenic acid, 15 mg; choline, 1000 mg; Zn, 100 mg; Fe, 120 mg; Cu, 150 mg; I, 0.3 mg; Se, 0.3 mg. ^2^ Content of digestible energy, crude protein, crude fat, crude fiber, Lys, Met + Cys, total P, and Ca were measured values, and others were calculated values.

**Table 2:** Essential microelements concentration in two kinds of diets (dry matter, ug/g).

Items	Diet 1 ^1^	Diet 2 ^2^	SEM	*p*-Value
Cu	79.40	63.04	4.434	0.060
Fe	453.23	452.85	7.356	0.981
Zn	365.20	341.54	9.560	0.207
Mn	112.24	91.13	6.441	0.102

SEM, standard error of mean. ^1^ Diet 1: basal diet. ^2^ Diet 2: basal diet supplemented with 2 mg/kg EGF (epidermal growth factor).

**Table 3:** Epidermal growth factor (EGF) attenuates the effect of lipopolysaccharide (LPS) on growth performance of early-weaning piglets.

Items	Treatment				Main Effect of L		Main Effect of E		SEM	*p*-Value			
	Control	EGF	LPS	EGF + LPS	Z_LPS_	H_LPS_	Z_EGF_	H_EGF_		Treatment	L	E	E × L
IBW, kg	7.92	7.84	7.65	7.65	7.88	7.65	7.78	7.74	0.089	0.675	0.247	0.844	0.834
FBW, kg	10.52	10.67	9.43	10.42	10.61	9.76	9.90	10.59	0.229	0.131	0.134	0.196	0.336
ADFI, g	264.54 ^a^	350.00 ^b^	319.34 ^b^	327.64 ^b^	321.51	322.11	301.07	342.55 ^§^	10.131	0.012	0.254	0.007	0.019
ADG, g	198.93	202.50	158.57	167.86	201.31 *	162.29	174.71	190.95	8.507	0.126	0.036	0.670	0.849
FCR, g/g	1.64	1.61	2.08	1.95	1.63	2.03 *	1.86	1.75	0.088	0.119	0.030	0.602	0.733

EGF, epidermal growth factor; LPS, lipopolysaccharide; L, LPS level; E, EGF level; E × L, interaction between EGF and LPS levels; H_LPS_, high LPS; Z_LPS_, low LPS; H_EGF_, high EGF; Z_EGF_, low EGF; IBW, initial body weight; FBW, final body weight; ADFI, average daily feed intake; ADG, average daily gain; FCR, feed conversion ratio; SEM, standard error of mean. ^a,b^ Values of groups in the same row with the same superscript or absence of a superscript were not significantly different (*p* > 0.05). * Values of main effects of L in the same row were significantly different (*p* < 0.05). ^§^ Values of main effects of E in the same row were significantly different (*p* < 0.05).

**Table 4:** EGF attenuates the effect of LPS on apparent nutrient digestibility in early-weaning piglets (dry matter).

Items	Treatment				Main Effect of L		Main Effect of E		SEM	*p*-Value			
	Control	EGF	LPS	EGF + LPS	Z_LPS_	H_LPS_	Z_EGF_	H_EGF_		Treatment	L	E	E × L
Crude protein	83.56	79.40	74.93	79.53	80.78	76.46	77.81	79.44	2.403	0.733	0.466	0.969	0.452
Crude fat	60.11 ^b^	60.17 ^b^	48.36 ^a^	58.28 ^b^	60.14 *	52.33	55.07	59.54	1.722	0.017	0.020	0.068	0.071
Crude fiber	49.49	44.00	51.30	47.04	46.36	49.60	50.40	45.01	1.801	0.501	0.540	0.233	0.876
Gross energy	83.89	82.17	80.67	82.55	83.03	81.29	82.05	82.32	1.507	0.912	0.697	0.982	0.624
P	64.68	58.31	58.70	58.91	60.86	58.77	60.70	58.55	1.593	0.609	0.469	0.410	0.381

EGF, epidermal growth factor; LPS, lipopolysaccharide; L, LPS level; E, EGF level; E × L, interaction between EGF and LPS levels; H_LPS_, high LPS; Z_LPS_, low LPS; H_EGF_, high EGF; Z_EGF_, low EGF; SEM, standard error of mean. ^a,b^ Values of groups in the same row with the same superscript or absence of a superscript were not significantly different (*p* > 0.05). * Values of main effects of L in the same row were significantly different (*p* < 0.05).

**Table 5:** EGF attenuates the effect of LPS on the concentration of Cu, Fe, Zn, Mn in the gastrointestinal tract chyme and feces of early-weaning piglets (dry matter, ug/g).

Items	Treatment				Main Effect of L		Main Effect of E		SEM	*p*-Value			
	Control	EGF	LPS	EGF + LPS	Z_LPS_	H_LPS_	Z_EGF_	H_EGF_		Treatment	L	E	E × L
Cu													
Stomach	46.72	49.06	49.65	45.40	48.06	47.10	47.89	47.49	0.692	0.092	0.754	0.427	0.020
Jejunum	57.59 ^a^	59.91 ^a^	91.36 ^b^	56.69 ^a^	58.75	68.24 *	71.10 ^§^	58.07	4.014	0.000	0.002	0.001	0.001
Ileum	183.85 ^c^	119.72 ^b^	179.97 ^c^	57.42 ^a^	145.37 *	103.38	181.52 ^§^	80.78	15.842	0.000	0.000	0.000	0.000
Feces	431.71	329.25	511.53	339.75	373.16	425.64	463.64 ^§^	332.75	27.625	0.051	0.305	0.012	0.424
Fe													
Stomach	317.02	328.78	327.66	312.51	321.72	320.08	321.28	320.64	5.056	0.719	0.816	0.888	0.294
Jejunum	330.67 ^a^	360.77 ^ab^	405.73 ^c^	391.30 ^bc^	348.73	398.52 *	368.20	372.98	10.548	0.017	0.004	0.490	0.088
Ileum	1333.46 ^c^	919.54 ^b^	1256.44 ^c^	675.66 ^a^	1057.52 *	966.05	1294.95 ^§^	838.25	83.487	0.001	0.027	0.000	0.180
Feces	2321.65 ^b^	1797.26 ^a^	2929.12 ^c^	1824.33 ^a^	2059.45	2376.72 *	2625.38 ^§^	1810.79	143.378	0.000	0.005	0.000	0.008
Zn													
Stomach	166.62	156.81	159.87	177.43	161.01	168.65	163.25	165.65	3.054	0.051	0.175	0.432	0.017
Jejunum	266.60	214.69	290.97	266.62	240.64	274.73 *	276.34 ^§^	244.36	10.276	0.054	0.042	0.042	0.408
Ileum	412.50 ^c^	138.89 ^a^	894.09 ^d^	281.11 ^b^	275.70	587.60 *	653.30 ^§^	210.00	85.836	0.000	0.000	0.000	0.000
Feces	1447.58 ^b^	1199.16 ^a^	1972.72 ^c^	1332.86 ^ab^	1298.53	1607.09 *	1762.67 ^§^	1275.56	93.526	0.000	0.001	0.000	0.019
Mn													
Stomach	38.94 ^a^	38.49 ^a^	45.11 ^b^	43.96 ^b^	38.72	44.53 *	42.64	41.77	1.001	0.001	0.000	0.290	0.631
Jejunum	179.48 ^b^	127.30 ^a^	190.53 ^b^	135.17 ^a^	153.39	162.85	185.00 ^§^	131.24	10.569	0.004	0.191	0.001	0.805
Ileum	275.11 ^c^	224.85 ^a^	283.15 ^c^	247.99 ^b^	249.98	265.57	279.13 ^§^	236.42	8.973	0.006	0.052	0.002	0.257
Feces	508.09 ^b^	389.36 ^a^	590.53 ^c^	414.04 ^a^	436.85	502.28 *	549.31 ^§^	399.23	28.673	0.000	0.007	0.000	0.064

EGF, epidermal growth factor; LPS, lipopolysaccharide; L, LPS level; E, EGF level; E × L, interaction between EGF and LPS levels; H_LPS_, high LPS; Z_LPS_, low LPS; H_EGF_, high EGF; Z_EGF_, low EGF; SEM, standard error of mean. ^a–d^ Values of groups in the same row with the same superscript or absence of a superscript were not significantly different (*p* > 0.05). * Values of main effects of L in the same row were significantly different (*p* < 0.05). ^§^ Values of main effects of E in the same row were significantly different (*p* < 0.05).

**Table 6:** EGF attenuates the effect of LPS on the expression levels of Cu transport-relative genes in the mucosa of gastrointestinal tract of early-weaning piglets.

Items	Treatment				Main Effect of L		Main Effect of E		SEM	*p*-Value			
	Control	EGF	LPS	EGF + LPS	Z_LPS_	H_LPS_	Z_EGF_	H_EGF_		Treatment	L	E	E × L
Ctr1													
Stomach	1.00	1.38	0.93	1.29	1.23	1.11	0.96	1.35 ^§^	0.087	0.157	0.594	0.043	0.960
Duodenum	1.00	1.13	1.06	1.33	1.07	1.19	1.03	1.21	0.065	0.431	0.381	0.186	0.598
Jejunum	1.00 ^b^	1.14 ^b^	0.52 ^a^	0.87 ^b^	1.08 *	0.73	0.76	1.01 ^§^	0.082	0.014	0.006	0.034	0.268
Ileum	1.00	1.01	0.79	0.87	1.01	0.85	0.90	0.93	0.054	0.550	0.183	0.702	0.768
Cox17													
Stomach	1.00 ^a^	3.53 ^c^	1.36 ^a^	2.49 ^b^	2.52	1.93	1.18	3.11 ^§^	0.372	0.000	0.077	0.000	0.006
Duodenum	1.00 ^b^	1.01 ^b^	0.45 ^a^	0.84 ^b^	1.01 *	0.60	0.67	0.92 ^§^	0.091	0.004	0.003	0.029	0.035
Jejunum	1.01	1.10	0.70	0.98	1.06	0.81	0.82	1.05	0.072	0.138	0.118	0.158	0.421
Ileum	1.00 ^b^	0.97 ^b^	0.35 ^a^	0.46 ^a^	0.99 *	0.41	0.68	0.72	0.116	0.013	0.003	0.696	0.489
Atox1													
Stomach	1.00 ^b^	1.81 ^c^	0.52 ^a^	0.71 ^a^	1.40 *	0.60	0.71	1.26 ^§^	0.174	0.000	0.000	0.001	0.006
Duodenum	1.00	0.91	0.83	0.96	0.96	0.91	0.93	0.94	0.026	0.154	0.215	0.652	0.044
Jejunum	1.00 ^b^	1.50 ^c^	0.58 ^a^	1.06 ^b^	1.30 *	0.82	0.75	1.28 ^§^	0.111	0.000	0.000	0.000	0.819
Ileum	1.00 ^b^	1.33 ^c^	0.88 ^a^	1.42 ^c^	1.16	1.15	0.94	1.38 ^§^	0.085	0.000	0.632	0.000	0.012
ATP7A													
Stomach	1.00 ^b^	1.89 ^c^	0.77 ^a^	2.02 ^c^	1.45	1.27	0.86	1.96 ^§^	0.196	0.000	0.322	0.000	0.015
Duodenum	1.00 ^b^	1.16 ^b^	0.59 ^a^	0.90 ^ab^	1.08 *	0.72	0.80	1.05 ^§^	0.078	0.014	0.009	0.042	0.427
Jejunum	1.00 ^a^	2.40 ^b^	1.35 ^a^	2.26 ^b^	1.56	1.74	1.20	2.31 ^§^	0.192	0.005	0.638	0.001	0.286
Ileum	1.00	0.94	0.99	0.95	0.97	0.97	1.00	0.95	0.031	0.925	0.988	0.556	0.902
ATP7B													
Stomach	1.00 ^a^	2.21 ^c^	1.49 ^b^	1.14 ^a^	1.49 *	1.31	1.20	1.68 ^§^	0.168	0.001	0.022	0.005	0.000
Duodenum	1.00 ^a^	1.52 ^b^	0.98 ^a^	1.16 ^a^	1.32 *	1.08	0.99	1.31 ^§^	0.067	0.000	0.006	0.000	0.010
Jejunum	1.00 ^a^	2.03 ^b^	1.01 ^a^	2.18 ^b^	1.62	1.59	1.00	2.09 ^§^	0.210	0.020	0.739	0.004	0.759
Ileum	1.00 ^b^	0.90 ^b^	0.42 ^a^	0.46 ^a^	0.94 *	0.44	0.65	0.68	0.083	0.000	0.000	0.551	0.224
CCS													
Stomach	1.00 ^a^	3.06 ^c^	0.83 ^a^	2.01b	2.03	1.42	0.92	2.53 ^§^	0.348	0.008	0.061	0.002	0.134
Duodenum	1.00	1.04	0.45	0.70	1.03 *	0.57	0.73	0.91	0.098	0.061	0.017	0.309	0.459
Jejunum	1.00 ^a^	2.34 ^c^	0.83 ^a^	1.77 ^b^	1.67 *	1.39	0.92	2.00 ^§^	0.206	0.000	0.001	0.000	0.014
Ileum	1.00 ^b^	0.96 ^b^	0.65 ^a^	0.69 ^a^	0.98 *	0.68	0.83	0.83	0.054	0.003	0.001	0.982	0.429

EGF, epidermal growth factor; LPS, lipopolysaccharide; L, LPS level; E, EGF level; E × L, interaction between EGF and LPS levels; H_LPS_, high LPS; Z_LPS_, low LPS; H_EGF_, high EGF; Z_EGF_, low EGF; Ctr1, copper transport protein1; Cox17, cytochrome c oxidase copper chaperone; Atox1, antioxidant 1; ATP7A, copper-transporting P-type 7A; ATP7B, copper-transporting P-type 7B; CCS, copper chaperone for superoxide dismutase; SEM, standard error of mean. ^a–c^ Values of groups in the same row with the same superscript or absence of a superscript were not significantly different (*p* > 0.05). * Values of main effects of L in the same row were significantly different (*p* < 0.05). ^§^ Values of main effects of E in the same row were significantly different (*p* < 0.05).

**Table 7:** EGF attenuates the effect of LPS on the expression levels of Fe transport-relative genes and DMT1 gene in the mucosa of gastrointestinal tract of early-weaning piglets.

Items	Treatment				Main Effect of L		Main Effect of E		SEM	*p*-Value			
	Control	EGF	LPS	EGF + LPS	Z_LPS_	H_LPS_	Z_EGF_	H_EGF_		Treatment	L	E	E × L
CYTB													
Stomach	1.00 ^a^	2.40 ^c^	1.59 ^b^	1.60 ^b^	1.84	1.60	1.30	2.08 ^§^	0.196	0.006	0.531	0.007	0.007
Duodenum	1.00 ^a^	1.08 ^ab^	0.71 ^a^	1.56 ^b^	1.05	1.28	0.86	1.36 ^§^	0.118	0.023	0.553	0.020	0.042
Jejunum	1.00 ^b^	1.11 ^b^	0.63 ^a^	0.58 ^a^	1.06 *	0.61	0.82	0.85	0.092	0.025	0.006	0.725	0.417
Ileum	1.00	1.52	0.99	1.42	1.31	1.16	0.99	1.48 ^§^	0.098	0.059	0.706	0.014	0.755
Hp													
Stomach	1.00 ^a^	2.02 ^b^	1.04 ^a^	2.57 ^c^	1.51	1.80	1.02	2.30 ^§^	0.257	0.003	0.090	0.001	0.119
Duodenum	1.05	1.20	0.86	1.17	1.15	1.01	0.96	1.19	0.103	0.739	0.650	0.380	0.758
Jejunum	1.00 ^a^	1.98 ^b^	1.60 ^ab^	2.03 ^b^	1.49	1.81	1.30	2.01 ^§^	0.170	0.049	0.154	0.018	0.213
Ileum	1.00 ^b^	1.17 ^bc^	0.45 ^a^	1.56 ^c^	1.09	1.00	0.72	1.37 ^§^	0.156	0.007	0.443	0.003	0.010
Tf													
Stomach	1.02 ^a^	3.29 ^c^	0.72 ^a^	2.21 ^b^	2.16 *	1.47	0.87	2.75 ^§^	0.390	0.001	0.014	0.000	0.078
Duodenum	1.01 ^b^	1.48 ^b^	0.28 ^a^	0.44 ^a^	1.29 *	0.34	0.57	1.07	0.177	0.002	0.001	0.060	0.300
Jejunum	1.01 ^ab^	1.26 ^b^	0.67 ^ab^	0.41 ^a^	1.16 *	0.54	0.84	0.92	0.133	0.043	0.014	0.968	0.170
Ileum	1.00	1.00	0.71	1.02	1.00	0.83	0.83	1.01	0.064	0.174	0.243	0.197	0.199
DMT1													
Stomach	1.00 ^a^	1.72 ^b^	1.05 ^a^	1.75 ^b^	1.43	1.40	1.03	1.73 ^§^	0.127	0.002	0.647	0.000	0.919
Duodenum	1.00 ^b^	2.42 ^c^	0.27 ^a^	0.35 ^a^	1.71 *	0.30	0.51	1.18 ^§^	0.255	0.000	0.000	0.000	0.000
Jejunum	1.04 ^b^	1.70 ^c^	0.43 ^a^	0.64 ^ab^	1.37 *	0.54	0.74	1.1 ^§^	0.189	0.010	0.004	0.034	0.174
Ileum	1.00 ^b^	0.89 ^b^	0.65 ^a^	0.91 ^b^	0.94 *	0.78	0.86	0.90	0.044	0.002	0.003	0.070	0.001

EGF, epidermal growth factor; LPS, lipopolysaccharide; L, LPS level; E, EGF level; E × L, interaction between EGF and LPS levels; H_LPS_, high LPS; Z_LPS_, low LPS; H_EGF_, high EGF; Z_EGF_, low EGF; CYTB, cytochrome b; Hp, hephaestin; Tf, transferrin; DMT1, divalent metal transporter 1; SEM, standard error of mean. ^a–c^ Values of groups in the same row with the same superscript or absence of a superscript were not significantly different (*p* > 0.05). * Values of main effects of L in the same row were significantly different (*p* < 0.05). ^§^ Values of main effects of E in the same row were significantly different (*p* < 0.05).

**Table 8:** EGF attenuates the effect of LPS on the expression levels of Mn transport-relative genes in the mucosa of gastrointestinal tract of early-weaning piglets.

Items	Treatment				Main Effect of L		Main Effect of E		SEM	*p*-Value			
	Control	EGF	LPS	EGF + LPS	Z_LPS_	H_LPS_	Z_EGF_	H_EGF_		Treatment	L	E	E × L
Zip4													
Stomach	1.01 ^a^	2.99 ^d^	1.46 ^b^	2.06 ^c^	1.80 *	1.70	1.24	2.53 ^§^	0.242	0.000	0.046	0.000	0.000
Duodenum	1.01	0.86	1.07	1.02	0.95	1.05	1.03	0.94	0.054	0.696	0.403	0.461	0.699
Jejunum	1.00 ^ab^	1.40 ^b^	0.73 ^a^	1.37 ^b^	1.27	1.05	0.84	1.39 ^§^	0.100	0.010	0.261	0.003	0.344
Ileum	1.01 ^b^	2.08 ^c^	0.46 ^a^	1.00 ^b^	1.54 *	0.73	0.68	1.43 ^§^	0.192	0.000	0.000	0.000	0.054
Zip7													
Stomach	1.02 ^c^	0.77 ^bc^	0.40 ^a^	0.64 ^ab^	0.85 *	0.52	0.71	0.73	0.073	0.007	0.002	0.961	0.016
Duodenum	1.01	1.25	0.57	0.98	1.15	0.73	0.75	1.14	0.112	0.088	0.079	0.099	0.616
Jejunum	1.01	1.09	1.24	0.75	1.05	1.00	1.12	0.92	0.077	0.108	0.647	0.118	0.050
Ileum	1.00 ^c^	0.82 ^b^	0.57 ^a^	0.67 ^ab^	0.91 *	0.61	0.74	0.74	0.062	0.006	0.002	0.426	0.036
ZnT1													
Stomach	1.00 ^a^	2.00 ^b^	1.38 ^a^	2.62 ^c^	1.50	2.00 *	1.19	2.31 ^§^	0.238	0.004	0.023	0.001	0.442
Duodenum	1.00	1.07	0.91	1.53	1.03	1.22	0.96	1.34	0.119	0.226	0.425	0.156	0.231
Jejunum	1.01	0.98	0.85	1.28	1.00	1.07	0.93	1.13	0.071	0.168	0.558	0.136	0.096
Ileum	1.00	1.15	1.04	1.38	1.08	1.24	1.02	1.29	0.077	0.248	0.374	0.126	0.504
ZnT4													
Stomach	1.00	1.17	1.01	1.14	1.10	1.07	1.01	1.16	0.064	0.785	0.936	0.363	0.919
Duodenum	1.01	1.16	0.94	1.30	1.07	1.08	0.97	1.23	0.068	0.301	0.791	0.094	0.467
Jejunum	1.00	0.92	1.09	1.06	0.96	1.07	1.05	0.99	0.046	0.677	0.326	0.595	0.833
Ileum	1.02	1.04	0.85	1.26	1.03	1.06	0.94	1.15	0.068	0.188	0.823	0.107	0.136

EGF, epidermal growth factor; LPS, lipopolysaccharide; L, LPS level; E, EGF level; E × L, interaction between EGF and LPS levels; H_LPS_, high LPS; Z_LPS_, low LPS; H_EGF_, high EGF; Z_EGF_, low EGF; Zip4, zrt-irt-like protein 4; Zip7, zrt-irt-like protein 7; ZnT1, zinc transporter 1; ZnT4, zinc transporter 4; SEM, standard error of mean. ^a–d^ Values of groups in the same row with the same superscript or absence of a superscript were not significantly different (*p* > 0.05). * Values of main effects of L in the same row were significantly different (*p* < 0.05). ^§^ Values of main effects of E in the same row were significantly different (*p* < 0.05).

## Data Availability

The data presented in this study are available on request from the corresponding author.

## Supplementary Materials

The following are available online at https://www.mdpi.com/article/10.3390/ani11061598/s1, Supplementary Table S1: The primers for quantitative real-time PCR.

## References

[1] Mukhopadhyay B.P. (2017). Recognition dynamics of trinuclear copper cluster and associated histidine residues through conserved or semi-conserved water molecules in human ceruloplasmin: The involvement of aspartic and glutamic acid gates. J. Biomol. Struct. Dyn..

[2] Alok G., Trivedi P.P., Timbalia S.A., Griffin A.T., Rahn J.J., Chan S.S.L., Gohil V.M. (2014). Copper supplementation restores cytochrome c oxidase assembly defect in a mitochondrial disease model of COA6 deficiency. Hum. Mol. Genet..

[3] Ridge P.G., Yan Z., Gladyshev V.N. (2008). Comparative genomic analyses of copper transporters and cuproproteomes reveal evolutionary dynamics of copper utilization and its link to oxygen. PLoS ONE.

[4] Hoes M.F., Beverborg N.G., Kijlstra J.D., Kuipers J., Swinkels D.W., Giepmans B.N.G., Rodenburg R.J., van Veldhuisen D.J., de Boer R.A., van der Meer P. (2018). Iron deficiency impairs contractility of human cardiomyocytes through decreased mitochondrial function. Eur. J. Heart Fail..

[5] Wijmenga C., Klomp L.W.J. (2004). Molecular regulation of copper excretion in the liver. Proc. Nutr. Soc..

[6] Ganz T., Aronoff G.R., Gaillard C.A.J.M., Goodnough L.T., Macdougall I.C., Mayer G., Porto G., Winkelmayer W.C., Wish J.B. (2020). Iron administration, infection, and anemia management in CKD: Untangling the effects of intravenous iron therapy on immunity and infection risk. Kidney Med..

[7] Goff J.P. (2018). Invited review: Mineral absorption mechanisms, mineral interactions that affect acid–base and antioxidant status, and diet considerations to improve mineral status. J. Dairy Sci..

[8] Joshi P., Bodnya C., Ilieva I., Neely M.D., Aschner M., Bowman A.B. (2019). Huntington’s disease associated resistance to Mn neurotoxicity is neurodevelopmental stage and neuronal lineage dependent. NeuroToxicology.

[9] Whiting T.L., Pasma T. (2008). Isolated weaning technology: Humane benefits and concerns in the production of pork. Can. Vet. J..

[10] Bedford A., Chen T., Huynh E., Zhu C., Medeiros S., Wey D., Lange C.D., Li J. (2015). Epidermal growth factor containing culture supernatant enhances intestine development of early-weaned pigs in vivo: Potential mechanisms involved. J. Biotechnol..

[11] Moeser A.J., Ryan K.A., Nighot P.K., Blikslager A.T. (2007). Gastrointestinal dysfunction induced by early weaning is attenuated by delayed weaning and mast cell blockade in pigs. Am. J. Physiol.-Gastrointest. Liver Physiol..

[12] Campbell J.M., Crenshaw J.D., Polo J. (2013). The biological stress of early weaned piglets. J. Anim. Sci. Biotechnol..

[13] Wang J.P., Yoo J.S., Lee J.H., Jang H.D., Kim H.J., Shin S.O., Seong S.I., Kim I.H. (2009). Effects of phenyllactic acid on growth performance, nutrient digestibility, microbial shedding, and blood profile in pigs. J. Anim. Sci..

[14] Hu C.H., Xiao K., Luan Z.S., Song J. (2013). Early weaning increases intestinal permeability, alters expression of cytokine and tight junction proteins, and activates mitogen-activated protein kinases in pigs. J. Anim. Sci..

[15] Sueyoshi K., Ledderose C., Shen Y., Lee A.H., Shapiro N.I., Junger W.G. (2019). Lipopolysaccharide suppresses T cells by generating extracellular ATP that impairs their mitochondrial function via P2Y11 receptors. J. Biol. Chem..

[16] George L., Ramasamy T., Sirajudeen K., Manickam V. (2019). LPS-induced apoptosis is partially mediated by hydrogen sulphide in RAW 264.7 murine macrophages. Immunol. Investig..

[17] Guo W., Liu B., Hu G., Kan X., Li Y., Gong Q., Xu D., Ma H., Cao Y., Huang B. (2019). Vanillin protects the blood-milk barrier and inhibits the inflammatory response in LPS-induced mastitis in mice. Toxicol. Appl. Pharm..

[18] He C., Deng J., Hu X., Zhou S., Wu J., Xiao D., Darko K.O., Huang Y., Tao T., Peng M. (2019). Vitamin A inhibits the action of LPS on the intestinal epithelial barrier function and tight junction proteins. Food Funct..

[19] Rui L., Song Z., Zhao J., Huo D., Fan Z., Hou D.X., Xi H. (2018). Dietary L -theanine alleviated lipopolysaccharide-induced immunological stress in yellow-feathered broilers. Anim. Nutr..

[20] Xu R. (1998). Bioactive peptides in milk and their biological and health implications. Food Rev. Int..

[21] Odle J., Zijlstra R.T., Donovan S.M. (1996). Intestinal effects of milkborne growth factors in neonates of agricultural importance. J. Anim. Sci..

[22] Nojiri T., Yoshizato T., Fukami T., Obama H., Yagi H., Yotsumoto F., Miyamoto S. (2012). Clinical significance of amphiregulin and epidermal growth factor in colostrum. Arch. Gynecol. Obstet..

[23] Cohen S. (1962). Isolation of a mouse submaxillary gland protein accelerating incisor eruption and eyelid opening in the new-born animal. J. Biol. Chem..

[24] Gregory H. (1975). Isolation and structure of urogastrone and its relationship to epidermal growth factor. Nature.

[25] Zeng F., Harris R.C. (2014). Epidermal growth factor, from gene organization to bedside. Semin. Cell Dev. Biol..

[26] Mehrabi M., Khodarahmi R., Shahlaei M. (2017). Critical effects on binding of epidermal growth factor produced by amino acid substitutions. J. Biomol. Struct. Dyn..

[27] Kim E., Leung H., Akhtar N., Li J., Barta J.R., Wang Y., Yang C., Kiarie E. (2017). Growth performance and gastrointestinal responses of broiler chickens fed corn-soybean meal diet without or with exogenous epidermal growth factor upon challenge with *Eimeria*. Poultry Sci..

[28] Weng M.S., Chang J.H., Hung W.Y., Yang Y.C., Chien M.H. (2018). The interplay of reactive oxygen species and the epidermal growth factor receptor in tumor progression and drug resistance. J. Exp. Clin. Cancer Res..

[29] Wang S., Lin Z., Chen H., Cao Y., Zhang Z., Yang J., Huang Y., Guo C. (2015). Analysis of the biological activities of *Saccharomyces cerevisiae* expressing intracellular EGF, extracellular EGF, and tagged EGF in early-weaned rats. Appl. Microbiol. Biotechnol..

[30] Kang P., Toms D., Yin Y.L., Cheung Q., Gong J., de Lange C., Li J.L. (2010). Epidermal growth factor-expressing *Lactococcus lactis* enhances intestinal development of early-weaned pigs. J. Nutr..

[31] Brand T.M., Iida M., Li C., Wheeler D.L. (2011). The nuclear epidermal growth factor receptor signaling network and its role in cancer. Discov. Med..

[32] Lee D.N., Chuang Y.S., Chiou H.Y., Wu F.Y., Yen H.T., Weng C.F. (2010). Oral administration recombinant porcine epidermal growth factor enhances the jejunal digestive enzyme genes expression and activity of early-weaned piglets. J. Anim. Physiol. Anim. Nutr..

[33] Xu X., Wang X.Y., Wu H.T., Zhu H.L., Liu C.C., Hou Y.Q., Dai B., Liu X.T., Liu Y.L. (2018). Glycine relieves intestinal injury by maintaining mTOR signaling and suppressing AMPK, TLR4, and NOD signaling in weaned piglets after lipopolysaccharide challenge. Int. J. Mol. Sci..

[34] Kang P., Zhang L.L., Hou Y.Q., Ding B.Y., Yi D., Wang L., Zhu H.L., Liu Y.L., Yin Y.L., Wu G.Y. (2014). Effects of L-proline on the growth performance, and blood parameters in weaned lipopolysaccharide (LPS)-challenged pigs. Asian-Australas. J. Anim. Sci..

[35] Cheung Q.C., Yuan Z., Dyce P.W., Wu D., Delange K., Li J. (2009). Generation of epidermal growth factor-expressing *Lactococcus lactis* and its enhancement on intestinal development and growth of early-weaned mice. Am. J. Clin. Nutr..

[36] Bedford A., Huynh E., Fu M., Zhu C., Wey D., de Lange C., Li J. (2014). Growth performance of early-weaned pigs is enhanced by feeding epidermal growth factor-expressing *Lactococcus lactis* fermentation product. J. Biotechnol..

[37] Zhou X., Zhang Y., He L., Wan D., Liu G., Wu X., Yin Y. (2017). Serine prevents LPS-induced intestinal inflammation and barrier damage via p53-dependent glutathione synthesis and AMPK activation. J. Funct. Foods.

[38] Xiong W., Ma H., Zhang Z., Jin M., Wang J., Xu Y., Wang Z. (2019). The protective effect of icariin and phosphorylated icariin against LPS-induced intestinal epithelial cells injury. Biomed. Pharmacother..

[39] Wang L., Zhu F., Yang H., Li J., Li Y., Ding X., Xiong X., Yin Y. (2019). Effects of dietary supplementation with epidermal growth factor on nutrient digestibility, intestinal development and expression of nutrient transporters in early-weaned piglets. J. Anim. Physiol. Anim. Nutr..

[40] Xu X., Liu Y., Tang M., Yan Y., Gu W., Wang W., Meng Q. (2018). The function of *Eriocheir sinensis* transferrin and iron in *Spiroplasma eriocheiris* infection. Fish Shellfish Immun..

[41] Sekler I., Sensi S.L., Hershfinkel M., Silverman W.F. (2007). Mechanism and regulation of cellular zinc transport. Mol. Med..

[42] Zheng D., Feeney G.P., Peter K., Hogstrand C. (2008). Regulation of ZIP and ZnT zinc transporters in zebrafish gill: Zinc repression of ZIP10 transcription by an intronic MRE cluster. Physiol. Genom..

[43] Pfalzer A.C., Bowman A.B. (2017). Relationships between essential manganese biology and manganese toxicity in neurological disease. Curr. Environ. Health Rep..

[44] Li L., Yang X. (2018). The essential element manganese, oxidative stress, and metabolic diseases: Links and interactions. Oxid. Med. Cell. Longev..

[45] Belyaeva E.A., Korotkov S.M., Saris N. (2011). In vitro modulation of heavy metal-induced rat liver mitochondria dysfunction: A comparison of copper and mercury with cadmium. J. Trace Elem. Med. Biol..

[46] Logeman B.L., Wood L.K., Lee J., Thiele D.J. (2017). Gene duplication and neo-functionalization in the evolutionary and functional divergence of the metazoan copper transporters Ctr1 and Ctr2. J. Biol. Chem..

[47] Oszvald M., Tömösközi S., Tamás L., Békés F. (2008). Role of rice and added wheat protein in the mixing properties of different rice flours. Acta Aliment. Hung..

[48] Hamza I., Prohaska J., Gitlin J.D. (2003). Essential role for Atox1 in the copper-mediated intracellular trafficking of the Menkes ATPase. Proc. Natl. Acad. Sci. USA.

[49] Da Silva E.S., Abril S.I.M., Zanette J., Bianchini A. (2014). Salinity-dependent copper accumulation in the guppy *Poecilia vivipara* is associated with CTR1 and ATP7B transcriptional regulation. Aquat. Toxicol..

[50] Zoller H., Theurl I., Koch R.O., Mckie A.T., Vogel W., Weiss G. (2003). Duodenal cytochrome B and hephaestin expression in patients with iron deficiency and hemochromatosis. Gastroenterology.

[51] Doguer C., Ha J., Gulec S., Vulpe C.D., Anderson G.J., Collins J.F. (2017). Intestinal hephaestin potentiates iron absorption in weanling, adult, and pregnant mice under physiological conditions. Blood Adv..

[52] McKie A.T. (2001). An iron-regulated ferric reductase associated with the absorption of dietary iron. Science.

[53] Fuqua B.K., Lu Y., Frazer D.M., Darshan D., Wilkins S.J., Dunn L., Loguinov A.V., Kogan S.C., Matak P., Chen H. (2018). Severe iron metabolism defects in mice with double knockout of the multicopper ferroxidases hephaestin and ceruloplasmin. Cell. Mol. Gastroenterol. Hepatol..

[54] Yu Y., Jiang L., Wang H., Shen Z., Cheng Q., Zhang P., Wang J., Wu Q., Fang X., Duan L. (2020). Hepatic transferrin plays a role in systemic iron homeostasis and liver ferroptosis. Blood.

[55] Parrow N.L., Li Y., Feola M., Guerra A., Casu C., Prasad P., Mammen L., Ali F., Vaicikauskas E., Rivella S. (2019). specificity of iron binding to transferrin modulates murine erythropoiesis and iron homeostasis. Blood.

[56] Zhang Y.N., Wang S., Huang X.B., Li K.C., Chen W., Ruan D., Xia W.G., Wang S.L., Abouelezz K., Zheng C.T. (2020). Estimation of dietary manganese requirement for laying duck breeders: Effects on productive and reproductive performance, egg quality, tibial characteristics, and serum biochemical and antioxidant indices. Poult. Sci..

[57] Gresakova L., Venglovska K., Cobanova K. (2016). Dietary manganese source does not affect Mn, Zn and Cu tissue deposition and the activity of manganese-containing enzymes in lambs. J. Trace Elem. Med. Biol..

[58] Wu Q., Mu Q., Xia Z., Min J., Wang F. (2021). Manganese homeostasis at the host-pathogen interface and in the host immune system. Semin. Cell Dev. Biol..

[59] Gunshin H., Mackenzie B., Berger U.V., Gunshin Y., Romero M.F., Boron W.F., Nussberger S., Gollan J.L., Hediger M.A. (1997). Cloning and characterization of a mammalian proton-coupled metal-ion transporter. Nature.

[60] Wang Z., Li X., Zhou B. (2020). *Drosophila* ZnT1 is essential in the intestine for dietary zinc absorption. Biochem. Biophys. Res. Commun..

[61] Reis B.Z., Vieira D.A.D.S., Maynard D.D.C., Silva D.G.D., Mendes-Netto R.S., Cozzolino S.M.F. (2020). Zinc nutritional status influences ZnT1 and ZIP4 gene expression in children with a high risk of zinc deficiency. J. Trace Elem. Med. Biol..

[62] Hara T., Takeda T.A., Takagishi T., Fukue K., Kambe T., Fukada T. (2017). Physiological roles of zinc transporters: Molecular and genetic importance in zinc homeostasis. J. Physiol. Sci..

[63] Chun H., Korolnek T., Lee C., Coyne H.J., Winge D.R., Kim B., Petris M.J. (2019). An extracellular histidine-containing motif in the zinc transporter ZIP4 plays a role in zinc sensing and zinc-induced endocytosis in mammalian cells. J. Biol. Chem..

[64] Adulcikas J., Norouzi S., Bretag L., Sohal S.S., Myers S. (2018). The zinc transporter SLC39A7 (ZIP7) harbours a highly-conserved histidine-rich N-terminal region that potentially contributes to zinc homeostasis in the endoplasmic reticulum. Comput. Biol. Med..

[65] Nishito Y., Kambe T. (2019). Zinc transporter 1 (ZNT1) expression on the cell surface is elaborately controlled by cellular zinc levels. J. Biol. Chem..

