# Supplementary material for: Dietary Supplementation of EGF Ameliorates the Negatively Effects of LPS on Early-Weaning Piglets: From Views of Growth Performance, Nutrient Digestibility, Microelement Absorption and Possible Mechanisms
Source: Animals (Basel). 2021 May 28;11(6):1598. doi: 10.3390/ani11061598 (PMC8227379; doi:10.3390/ani11061598)
Supplement: Supplementary file 1 [file animals-11-01598-s001.zip › animals-1233996-supplement.pdf]

Supplementary Table S1

The primers for quantitative real-time PCR.

Gene	Primer sequence (5′ to 3′)	Accession number	Size, bp
Zip4	F: TGGCTGTGTATGGGCTGTCT<br>R: ACTGGCTGAGCTGGTCCTG	JF346412.1	141
Zip7	F: TCCAGGCATCAAGCAAGA<br>R: CCACCCGAAGCAAAACT	NM_001131045.1	171
ZnT1	F: AACCGACCAGGAGGAGAC<br>R: TACTACAATCACGGAACCCA	FJ374262.1	244
ZnT4	F: GTGGACCCCTGTGACAACTG<br>R: CTGACAAGACCTCTAAGCGATG	EU835903.1	277
Ctr1	F: CCTATGACCTTCTACTTTGGCTT<br>R: CGGGCTATCTTGAGTCCTTC	AF320815.2	137
Cox17	F: CTGAATCGCAGGAGAAGAAG<br>R: TGGGCCTCAATTAGGTGTC	NM_001348525.1	121
Atox1	F: CTCTAACGCAGTCACTCGGG<br>R: CAGACCTTCTTGTTGGGCA	NM_001167641.2	79
ATP7A	F: CAGGAGTAGGTGCTCAAAATG<br>R: ATGGGTAATGGTTCCAGTCTT	AB271958.1	107
ATP7B	F: GAACCCCAAGGCTCATCAC<br>R: CCGAGGAACTGGACAAAGG	AB271959.1	228
CCS	F: CGATGAGGGAGAAGACGACC<br>R: AGCGAGCGATGATGCCA	AY573056.1	98
DMT1	F: TCTTATGAGCATTGCCTACCT<br>R: AACCTTGGGATACTGACGG	EU647217.1	199
CYTB	F: TACTTATGGGAGTGACCGAGA<br>R: GACTATCCAAAAAATGAGAGCC	AM268434.1	137
Hp	F: ATCTCCCACCATAACCTCACC<br>R: CCACCTCCTGTTTCTTTCCC	NM_214000.2	157
Tf	F: AAACAGTGGTGGGAAAATAGAG<br>R: CCGCAATGTAGATGTAGCCT	NM_001244653.1	119
GAPDH	F: ATTCCACGGCACAGTCAA<br>R: AGCAGAAGGGGCAGAGAT	AF017079.1	214
